# Supplementary material for: Development of American-Lineage Influenza H5N2 Reassortant Vaccine Viruses for Pandemic Preparedness
Source: Viruses. 2019 Jun 11;11(6):543. doi: 10.3390/v11060543 (PMC6631248; doi:10.3390/v11060543)
Supplement: Supplementary file 1 [file viruses-11-00543-s001.pdf]

Figure S1. Growth curve of E7-V15 C11 virus.

The growth curve was conducted in Vero cells with 0.0001 MOI.

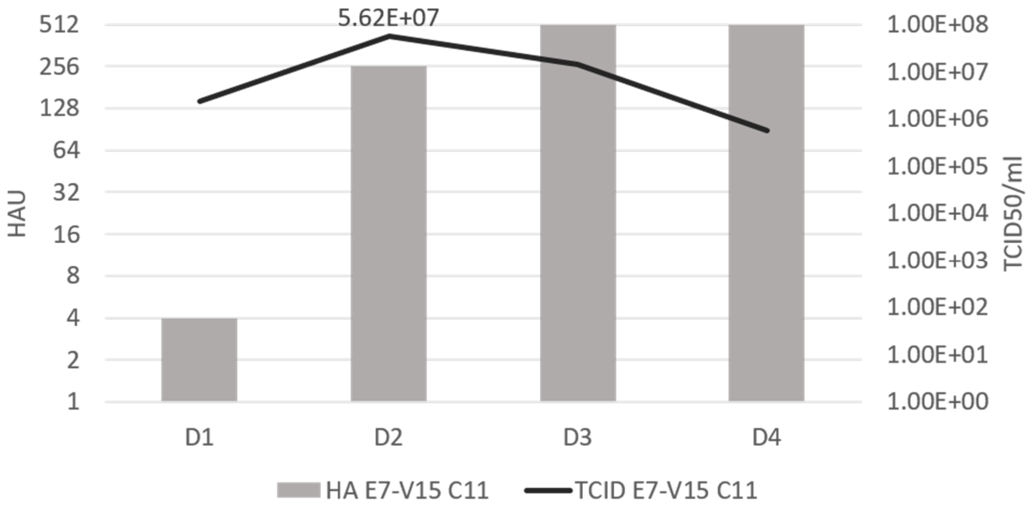

Figure S2. Trypsin dependency test of the H5N2 reassortant virus, E7-V15 C11.

Trypsin dependency test was conducted by plaque assay with trypsin-contained agarose or agarose without trypsin.

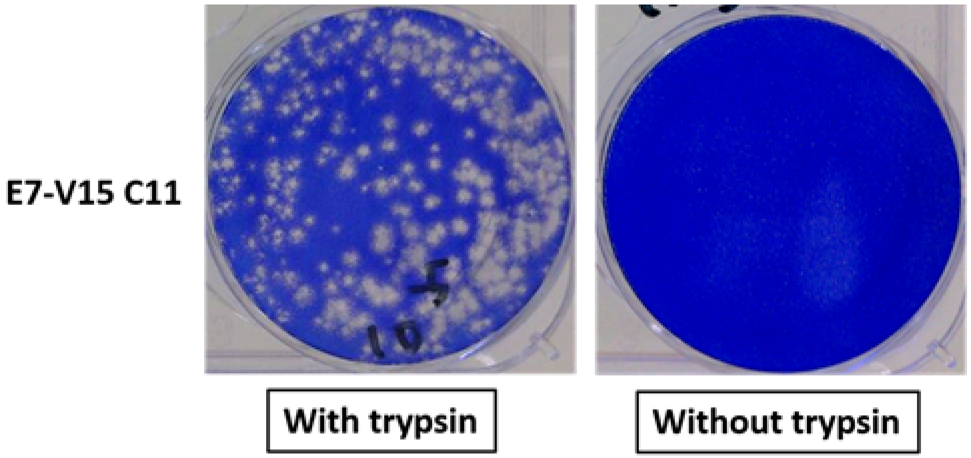

Figure S3. The change of body weight and temperature in pathogenicity assay in ferrets.

Four ferrets were intranasally inoculated with the vaccine virus, E7-V15 C11. The body weight and temperature were measured within the first 14 days after inoculation. The solid and dashed lines represent body temperature and body weight changes, respectively.

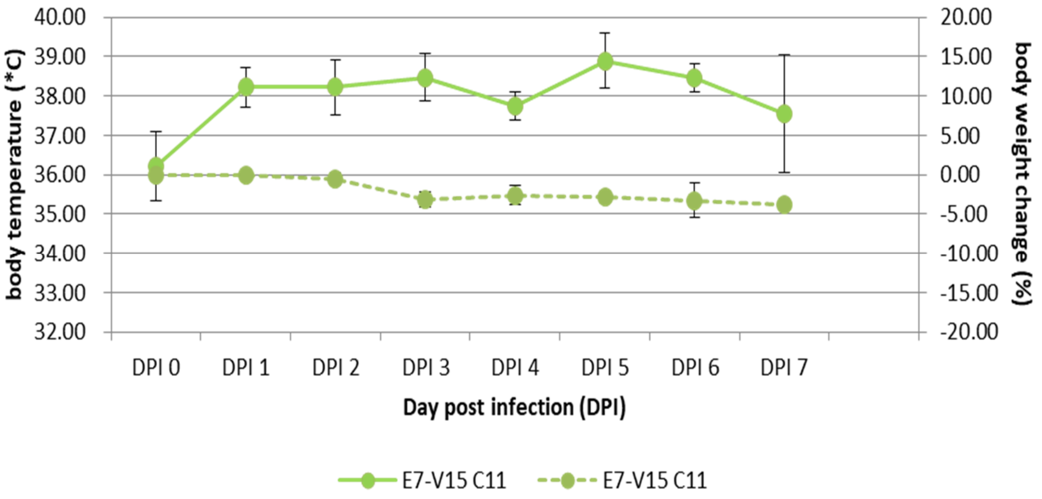

Figure S4. The HA content of purified virus, E7-V15 C11, was analysed by using SDS-PAGE.

Each lane contained 3 μg of purified virus. Lane 1: non-reduced sample; Lane 2: reduced sample; Lane 3-5: sample treated with PNGase (deglycosylation); Lane 6-8: resolved freeze-dry sample and treated with PNGase; Lane 9: PNGase only. The bands of virus proteins were verified by LC-MS/MS.

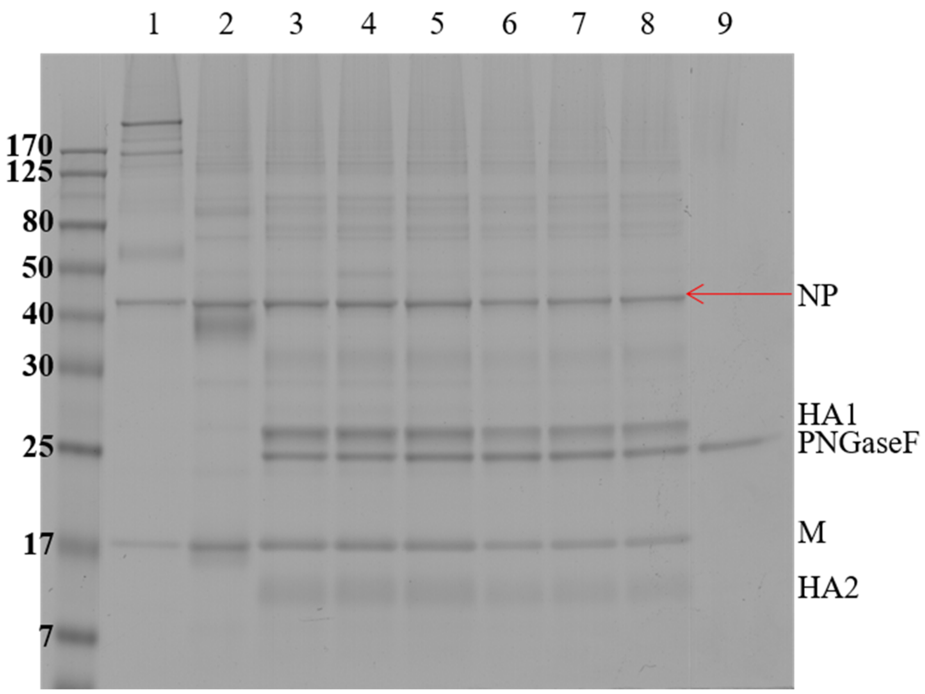

Table S1. The cross-reactive HI antibody titres using Eurasian-lineage H5N1 mouse anti-sera.

	Mouse sera	
**virus**	**rRG6[a]** **(H5N1 clade 2.3.4)**	**Vero-adapted NIBRG14** **(Vero-15)** **(H5N1 clade 1)**
A/CK/CY/A2628/2012 (E7)	<20	<20
A/CK/YL/0502/2012 (R3)	<20	20
RG6	160	<20
NIBRG14	<20	320

a: The reassortant H5N1 vaccine virus, rRG6 (A/Anhui/1/05 x PR8), was generated using reverse genetics in our laboratory.

Table S2. The sequence of the primers used in this study

Bm-HA-F	TATTCGTCTCAGGGAGCAAAAGCAGGGG	Hoffmann E et al., 2001, Arch Virol
Bm-NS-R	ATATCGTCTCGTATTAGTAGAAACAAGG GTGTTTT	Hoffmann E et al., 2001, Arch Virol
Bm-NA-F	TATTCGTCTCAGGGAGCAAAAGCAGGAG T	Hoffmann E et al., 2001, Arch Virol
Bm-NA-R	ATATCGTCTCGTATTAGTAGAAACAAGG AGTTTTTT	Hoffmann E et al., 2001, Arch Virol
08-forward	CTGAGAAACATACCCCAAGGAGAAACA AGAGGCCTATTTGGAGC	This study
08-reverse	GCTCCAAATAGGCCTCTTGTTTCTCCTTG GGGTATGTTTCTCAG	This study
H5 (H5N2)-670F	CAGAGGTCAATCCCAGAAATAGC	This study
H5 (H5N2)-900R	CATTGTGGAAAGGCATACTGGAA	This study
N2 (H5N2)-600F	GATGCTCGTTGACAGTATAGGTTC	This study
N2 (H5N2)-800R	GTCTCTGCATACACATCTGACATT	This study
PB2-1F	AGCGAAAGCAGGTCAATTATATT	This study
PB2-1R	GGCACATCTCCAATAAAGATGC	This study
PB2-2F	ACATAGTGAGAAGAGCTGCAGTATC	This study
PB2-2R	CCAGTTTCTGATGATCCATTGA	This study
PB2-3F	GATTAATGGTCCTGAATCAGTGTT	This study
PB2-3R	AGTAGAAACAAGGTCGTTTTTAAACT	This study
PB1-1F	AGCGAAAGCAGGCAAACC	This study
PB1-1R	GCCAACTTTGCTTTCTTCTCAT	This study

PB1-2F	TTGAACAATCAGGGTTGCC	This study
PB1-2R	ATGAACAACTGAAGGGCCA	This study
PB1-3F	AATATGATAAACAATGATCTTGGTCC	This study
PB1-3R	AGTAGGAACAAGGCATTTTTTCA	This study
PA-1F	AGCGAAAGCAGGTACTGATCC	This study
PA-1R	GCTTGCCCTCAATGTAGCC	This study
PA-2F	TAGAGCCTATGTGGATGGATTC	This study
PA-2R	CCCTCCTTAGTTCTACACTTGCT	This study
PA-3F	TGCCTTACTTAATGCATCTTGTG	This study
PA-3R	AGTAGAAACAAGGTACTTTTTTGGAC	This study
NP-1F	AGCAAAAGCAGGGTAGATAATCA	This study
NP-1R	TGCAGACCGTGCTAGAAAAGT	This study
NP-2F	CAAGTGAGAGAGAGCCGGG	This study
NP-2R	AGTAGAAACAAGGGTATTTTTCTTTAAT	This study
NS-1F	AGCAAAAGCAGGGTGACAA	This study
NS-1R	AGTAGAAACAAGGGTGTTTTTTATTAT	This study
M1-1F	AGCGAAAGCAGGTAGATATTGA	This study
M1-2R	AGTAGAAACAAGGTAGTTTTTTACTCC	This study

Table S3. The antigenicity of reassortant viruses analyzed using HI assay.

	Ferret sera			Chicken sera[a]	Goat serum[b]
virus	**R3**	**CH/2003**	**E7-V15 C11[c]**	**E7-V15 C11**	**E7 bHA**
A/CK/CY/A2628/2012 (E7)	160, 320 (226)	320, 320 (320)	80, 160 (113)	80, 160 (113)	320
A/CK/YL/0502/2012 (R3)	80, 160 (113)	160, 160 (160)	40, 160 (80)	40, 80 (56)	160
E7-V15 C11	80, 80 (80)	160, 160 (160)	160, 320 (226)	80, 160 (113)	320

a: The background information of chicken anti-(E7-V15 C11) sera were described in Table S4 (serum no. 3 and 8).

b: Goat anti-E7 bHA serum was standard serum generated in this study (result 3.6)

c: The background information of ferret anti-(E7-V15 C11) sera were described in Table 3 (ferret serum no. 265 and 266).

Table S4. The HI titer of E7-V15 C11 virus-infected chickens.

Nine chickens were used for pathogenicity test. After 10 days monitor, the chickens were sacrificed and the sera were taken for HI assay.

**No.**	**1**	**2**	**3**	**4**	**5**	**6**	**7**	**8**	**9**
HI titer	32	8	128	64	16	16	16	128	32

Table S5. Virus titers (log (TCID50)) of organs from infected ferrets were tested with Vero cells.

Each group having four ferrets were infected with the vaccine virus, E7-V15 C11. Two ferrets in the group were sacrificed on the third day after infection and the rest two on the 14th day. NT: nasal turbinate; UpRT: upper respiratory tract (top 1/3 of the trachea); LoRT: lower respiratory tract (bottom 1/3 of the trachea); HLN: Hilar lymph node; M: male; F: female.

	Vaccine virus (E7-V15 C11)			
No. of ferret	239 (M)	240 (F)	238 (M)	241 (F)
Sacrifice at	Day 3		Day 14	
NT	<1	<1	<1	<1
UpRT	<1	<1	<1	<1
LoRT	<1	<1	<1	<1
HLN	<1	<1	<1	<1
Lung	<1	<1	<1	<1
Brain	<1	<1	<1	<1
Homologous HI titer	-	-	20	20

Table S6. The HA content and HA:NP ratio of the E7-V15 C11 purified whole virus antigens.

	**E7-V15 C11**
**TOTAL PROTEIN (MG/ML) BASED ON LOWRY ASSAY**	177.77
**HEMAGGLUTININ RATIO (%) BASED ON SDS-PAGE**	36.4
**HEMAGGLUTININ CONTENT (MG/ML) BASED ON SDS-PAGE**	64.8
**HA/NP RATIO BASED ON SDS-PAGE**	1.45
